# Formulation and Characterization of Gelatin-Based Hydrogels for the Encapsulation of *Kluyveromyces lactis*—Applications in Packed-Bed Reactors and Probiotics Delivery in Humans

**DOI:** 10.3390/polym12061287

**Published:** 2020-06-04

**Authors:** Jorge Luis Patarroyo, Juan Sebastian Florez-Rojas, Diego Pradilla, Juan D. Valderrama-Rincón, Juan C. Cruz, Luis H. Reyes

**Affiliations:** 1 Grupo de Diseño de Productos y Procesos (GDPP), Department of Chemical Engineering, Universidad de los Andes, Bogotá, DC 111711, USA; jl.patarroyoa@uniandes.edu.co (J.L.P.); js.florez11@uniandes.edu.co (J.S.F.-R.); pradil@uniandes.edu.co (D.P.); 2 Department of Environmental Engineering, Universidad Antonio Nariño, Bogotá, DC 111711, USA; juan.d.valderrama@gmail.com; 3 Department of Biomedical Engineering, Universidad de los Andes, Bogotá, DC 111711, USA

**Keywords:** hydrogels, gelatin matrix, crosslinking, probiotics, encapsulation

## Abstract

One of the main issues when orally administering microorganism-based probiotics is the significant loss of bioactivity as they pass through the gastrointestinal (GI) tract. To overcome these issues, here, we propose to encapsulate the probiotic yeast *Kluyveromyces lactis* on chemically crosslinked gelatin hydrogels as a means to protect the bioactive agents in different environments. Hydrogels were prepared by the chemical crosslinking of gelatin, which is commercially available and inexpensive. This is crucial to ensure scalability and cost-effectiveness. To explore changes in key physicochemical parameters and their impact on cell viability, we varied the concentration of the crosslinking agent (glutaraldehyde) and the gelatin. The synthesized hydrogels were characterized in terms of morphological, physical-chemical, mechanical, thermal and rheological properties. This comprehensive characterization allowed us to identify critical parameters to facilitate encapsulation and enhance cell survival. Mainly due to pore size in the range of 5–10 μm, sufficient rigidity (breaking forces of about 1 N), low brittleness and structural stability under swelling and relatively high shear conditions, we selected hydrogels with a high concentration of gelatin (7.5% (w/v)) and concentrations of the crosslinking agent of 3.0% and 5.0% (w/w) for cell encapsulation. Yeasts were encapsulated with an efficiency of about 10% and subsequently tested in bioreactor operation and GI tract simulated media, thereby leading to cell viability levels that approached 95% and 50%, respectively. After testing, the hydrogels’ firmness was only reduced to half of the initial value and maintained resistance to shear even under extreme pH conditions. The mechanisms underlying the observed mechanical response will require further investigation. These encouraging results, added to the superior structural stability after the treatments, indicate that the proposed encapsulates are suitable to overcome most of the major issues of oral administration of probiotics and open the possibility to explore additional biotech applications further.

## 1. Introduction

Attention towards the consumption of functional foods in the general public has shifted because of the different long-term health benefits [1]. This is partly due to the incorporation of bioactive agents (e.g., omega-3 fatty acids, minerals, vitamins, proteins, peptides, probiotics, fiber and prebiotics) with proven activity towards mitigating impaired cellular functions. These bioactive agents have been associated with different conditions, including cancer, diabetes, cardiovascular disease (CVD), hypertension, diarrhea, lactose intolerance and some allergies [2,3]. Of particular interest are probiotics, mainly due to their antimutagenic, anticarcinogenic, anti-infection, immunomodulatory and cholesterol reduction properties [2,4].

Widely used probiotics include microorganisms, such as *Lactobacillus* and *Bifidobacterium* and yeast, such as *Saccharomyces boulardii* [5]. They are conventionally administered orally in preparations with concentrations between 10^6^ and 10^9^ CFU/mL. Additionally, the preparations include excipients such as microcrystalline cellulose, rice maltodextrin, silicon dioxide, magnesium stearate and hydroxypropyl methylcellulose [6]. These molecules serve as binders, diluents, lubricants and gliding, anti-caking, dispersants and viscosity-enhancing agents [7]. The incorporation of these species increases the production costs, which ultimately impacts the final price.

One of the most challenging issues during functional food manufacturing is to ensure that the active components can maintain their structural stability during storage and consumption [8]. This is mainly due to their pass through the gastrointestinal (GI) tract where the pH of the environment continually changes, and enzyme activity may negatively impact these components [9]. Different strategies have been developed to overcome this issue, which includes freeze and spray drying, emulsions, microencapsulation, nanoencapsulation and encapsulation in polymeric matrices [2,10,11]. Moreover, by controlling the parameters of encapsulation, it is possible to maintain relatively high cell viability and stability at both the culture and storage stages [12]. Despite some success cases over the past few years, issues regarding material integrity as it passes through the GI tract are yet to be solved. This is problematic because a lower amount of the probiotic reaches the site of action [8]. For this reason, considerable effort is still needed to ensure that the oral delivery of probiotics reaches a higher commercial success.

Hydrogels are usually defined as versatile materials with the ability to incorporate water into their three-dimensional network without losing integrity [13]. This feature is essential for different biological, biomedical and biotechnological processes, where the hydrogels need to be immersed in liquid media. One aspect of concern about the use of hydrogels is to properly tune their mechanical properties according to the intended application [14,15]. This has been achieved by implementing different strategies, including enzymatic crosslinking, physical crosslinking, chemical modifications and chemical crosslinking [15]. As discussed by Saez et al. [13], the strength of covalent bonds generated by chemical crosslinking may be able to provide the mechanical resistance that is needed for biomedical applications, including tissue engineering and drug delivery [14]. Hydrogels are, however, subject of instability and macroscopic deformation, particularly when subjected to a considerable degree of swelling [13]. These attributes have been crucial for the development of several applications, including industrial biotransformation, antimicrobial peptide production, cell encapsulation, water and air purification and medical applications such as tissue engineering and cell therapy [14,16,17,18,19,20,21,22,23,24,25,26,27,28,29,30].

The protection provided by hydrogels to encapsulated microorganisms has been crucial for applications in bioremediation and metabolite production [31,32]. This has been the case due to their incorporation as packing materials into highly efficient bioreaction systems. Some of the preferred species include *Saccharomyces*, *Kluyveromyces* and *Lactobacillus* [16,33,34]. Bubble columns and both concentric-tube and external-loop airlift bioreactors have been extensively used [35,36,37,38]. With this approach, it has been possible to produce numerous metabolites of commercial interest such as bioethanol, cellulose, biohydrogen, oxalic acid, gluconic acid, citric acid, malic acid and lactic acid [35,36,37,39,40]. Additionally, it has been possible to improve the quality of wastewaters by reducing the contents of contaminants, such as heavy and cationic metals, phenol and dyes [41,42]. An exciting example of bioremediation has been recently reported where crosslinked chitosan hydrogels were used to prepare capsules of *S. cerevisiae* to recover Europium and other precious lanthanides [43].

Due to its biological origin, low cytotoxicity, non-immunogenicity, biodegradability, ease of functionalization and inexpensiveness, gelatin has been extensively used as an encapsulating agent for ethyl benzoate [44], plasmid DNA material [45], sulphamethoxazole and clove oil [46] apart from several cells types [15,28,47]. Gelatin is a protein-based material derived from the hydrolysis of collagen. It can be classified as either type A or B, depending on the pretreatment through which it was obtained. In type A, the processing conditions are acidic to reach an isoelectric point between pH 8 and 9, whereas, for type B, it is obtained under alkaline treatment getting an isoelectric point between 4.5 and 5.6 [48]. The primary amino acids in the gelatin chains, which are almost 55% of the total contents, are glycine, proline, hydroxyproline and glutamic acid [48]. To ensure sufficient mechanical stability and long-term integrity, the manufacturing of collagen matrices must include a crosslinking agent for gelatin and macromolecules [15].

Bacterial strains such as *L. acidophilus* La-5, *L. casei* Shirota, *L. rhamnosus* GG, *L. johnsonii* NCC 533 [34] and *Bacillus subtilis* and *B. breve* [12] have been used as encapsulation agents for probiotics. Some of the applications include the preparation of food products such as fermented milk, cheese, ice cream, fermented meats such as sausages, desserts, confectionery, dietary supplements and drinks [49,50]. In the particular case of dairy products, other than bacteria, yeast strains have also been used due to their ability to metabolize lactose. An enthralling example is *K. lactis,* which produces the lactase enzyme, widely used for the manufacture of milk-based products aimed to lactose-intolerant individuals [51]. The ability to incorporate lactose through its wall cell is given by the lactose permease that is produced by the LAC12 gene and then decomposed to glucose and galactose by *β*-galactosidase that is encoded by the LAC4 gene [52,53]. Moreover, *K. lactis* has the GRAS (generally recognized as safe) status given by the U.S. Food and Drug Administration (FDA) [54], which allows the bioactive food industry to take advantage of its probiotic potentials, such as improving intestinal barrier function and enhancing immune functional activities [55]. Finally, *K. lactis* has also been utilized for recombinant protein expression, secreted and intracellular enzyme production at industrial scale and secondary metabolite production with high yields [51,56].

This work is therefore dedicated to encapsulating the yeast strain *K. lactis* into gelatin type-A hydrogels to produce low-cost and highly stable probiotic vehicles. The capsules were created by covalent crosslinking of glutaraldehyde with the polymer. The thermal, rheological and mechanical properties, as well as the microscopic characteristics of the prepared matrices, were evaluated before the encapsulating step. Upon encapsulation, successful proof-of-concept experiments were performed on a milliliter-scale bioreactor and a simulated gastrointestinal medium, where yields, cell viability and biocompatibility were determined. The manufactured encapsulated product has the potential to be considered for oral delivery of therapeutics and cells that exhibited limited stability under the conditions of the GI tract.

## 2. Materials and Methods

### 2.1. Microorganisms and Culture Media

The microorganism selected for encapsulation was *Kluyveromyces lactis* GG799 wild type from *K. lactis* Protein Expression Kit (New England Biolabs, Ipswich, MA, USA). It was maintained in YPGlu plates [yeast extract 1.0% (w/v), peptone 2.0% (w/v), glucose 2.0% (w/v), agar 1.5% (w/v), ampicillin 100 ug/mL] and inoculated in YNB liquid medium [yeast nitrogen base (YNB) 0.68% (w/v), glucose 2.0% (w/v), lactose 2.0% (w/v), L-histidine 0.001% (w/v)] [57]. To ensure sterility, the YPGlu medium was autoclaved and the YNB was filtered because of its thermolabile components. The inoculum was incubated in an orbital shaker at 30 °C and 200 RPM for 16 h. Subsequently, the culture was centrifuged at 4700 RPM for 10 min at 4 °C. The supernatant was discarded, and the final total mass of dried biomass registered [58]. Yeast cells were washed twice and resuspended with sterile water and stored at 4 °C for further use.

The fermentation medium (for bioreactor application) was YNB enriched with lactose (16% (w/v)) [33]. Growth kinetics were measured by incubating a single colony in YNB liquid medium at 30 °C and 200 RPM overnight. The culture OD_600_ was registered for two days by triplicate [58].

### 2.2. Preparation of Gelatin Hydrogels

Sterile milli-Q water was heated to 40 °C and mixed with gelatin Type A (food grade). The mixture was kept under constant stirring at 180 RPM for 30 min until a homogeneous mixture was achieved. Glutaraldehyde (GTA) solution of 25% for synthesis (PanReac AppliChem, Barcelona, Spain) was added dropwise while stirring at 80 RPM (to ensure complete chemical crosslinking) in a water bath at 40 °C for 2 h, maintaining the pH in a range between 6.5 and 6.8. The crosslinking process involved the reaction of free amine groups of lysine and hydroxylysine from the collagen, with the aldehyde groups of glutaraldehyde, as shown in Figure S1 (in Supplementary Materials). The primary amines react with aldehydes to form imine bonds [48]. The mixture was cooled down to room temperature and poured into silicone molds. Finally, the hydrogels were stored at 4 °C for 24 h before any test.

### 2.3. Probiotic Encapsulation

**Figure 1:**
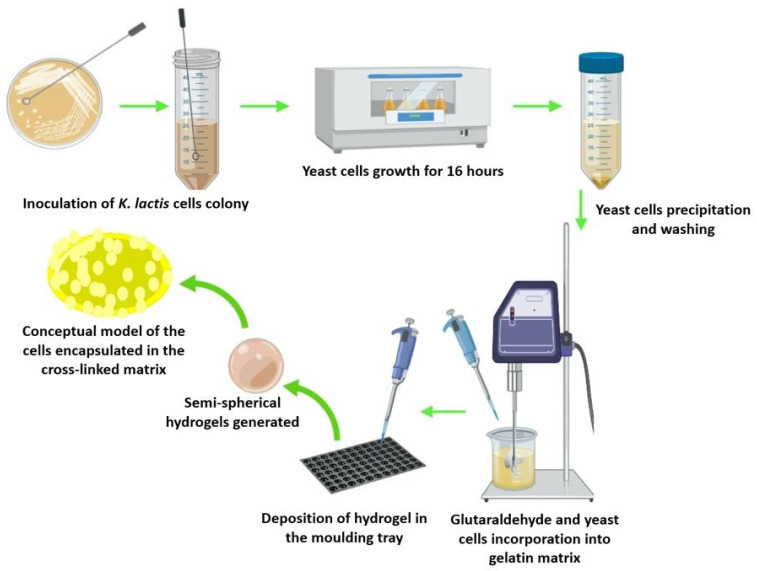
The protocol followed for the encapsulation of probiotic cells in the gelatin- glutaraldehyde (GTA)matrix.

After the incorporation of glutaraldehyde to the hydrogels, the yeast cells were resuspended in sterile water and carefully poured into the hydrogel solution to 5.0% (w/v). The process was conducted under low speed stirring for 30 min to avoid cell disruption. The mixture was cooled down to room temperature, deposited into sterilized silicone molds and sealed to prevent contamination. The hydrogels were stored at 4 °C for 24 h to complete the gelation process. This procedure was performed in a laminar flow hood, using sterilized materials and equipment. The protocol for the encapsulation of the probiotic is shown in Figure 1.

The cell encapsulation efficiency (EE) was calculated using Equation (1). Collagenase (Sigma-Aldrich, Saint Louis, MO, USA) was used to digest the gel’s peptide bonds. This was accomplished by placing a single hydrogel in 0.2% (w/v) collagenase, 0.36 mM CaCl_2_ (enzyme cofactor), YNB medium and incubated at 30 °C and 200 RPM for 17 h. The solution was then centrifuged at 1200 RPM and 4 °C for 10 min to collect the cells. The supernatant was discarded, and the pellet was resuspended in PBS solution. Suitable dilutions were used for sowing in YPGlu medium with agar. These cell cultures were incubated at 30 °C and finally, the Colony Forming Units (CFU) were counted.

(1) $$EE=\frac{CFU_{digested\;hydrogel}}{CFU_{initial\;cell\;culture}}\cdot 100.$$

### 2.4. Experimental Design

For characterization purposes, a $3^{2}$ experimental design (2 factors with three levels each) was used. The factors evaluated were the concentrations of gelatin and glutaraldehyde (GTA) in the hydrogel. The three gelatin concentrations selected for this study were 3.0%, 5.0% and 7.5% (w/v) while those of glutaraldehyde (with respect to gelatin) were 0.0%, 1.0%, 3.0% and 5.0% (w/w). The selected concentrations were also previously reported for gelatin hydrogels [59]. The probiotic yeast cells encapsulation proceeded according to the results of the thermal, mechanical, rheological, morphological and compositional characterization of the hydrogels (see below for details).

### 2.5. Survival Rate of Encapsulated Probiotics

The probiotic cells were stained with a fluorescent marker, observed under a confocal microscope and counted to estimate the live/dead ratio. These analyses were carried out for the hydrogels after packed bioreactor operation and GI tract treatments. Thin cross-sections (about 2 mm thick) were cut off each hydrogel, washed with PBS [19]. Finally, a propidium iodide solution (as a fluorescent marker) was added on the gel surface for staining. The gel cross-sections were kept in darkness for 30 min to let the marker diffuse into the porous hydrogel. Propidium iodide stains red those cells that have compromised membranes (i.e., dead cells) [60]. Finally, image acquisition was performed in a Confocal Laser Scanning Microscope Olympus FV1000 (40×, 0.6 NA) and the live/dead ratio was calculated by processing images with the aid of the Fiji-ImageJ software [61].

### 2.6. Morphological Structure and Beads Conformation

The structure and morphology of the prepared hydrogels were observed with the JEOL scanning electron microscope (model JSM 6490-LV). The observation was performed directly on a cooling stage at −15 °C with a liquid nitrogen-mediated fracturing setup to avoid alterations of the gel surface. High-resolution images were obtained at 1000× and 3000× magnification (10 kV). These images were used to determine the average pore size of each hydrogel with the aid of the software ImageJ [62]. Additionally, hydrogels with encapsulated probiotic yeast cells were observed to verify their matrix fixation and changes in the pore size, both before and after bioreactor operation.

### 2.7. Spectroscopy Analyses

Chemical bonding and functional groups were analyzed via non-destructive near (NIR) and far-infrared (IR) spectroscopy. NIR spectra were collected with a NIRS 5000 (FOSS, Hilleroed, Denmark) in the range of 800 to 2500 nm. Fourier Transform Infrared (FTIR) spectra were obtained by ALPHA-Eco ATR Spectrometer FT-IR (Bruker, Billerica, MA, USA) in the range of 4000 to 600 cm^−1^ by averaging three scans with 2 cm^−1^ resolution. Water was used as a reference. Samples were analyzed with both instruments with no prior treatment or addition of extra reagents.

### 2.8. Swelling Percentage Determination

Hydrogels swelling was studied by submerging them in an aqueous medium at a physiological pH of 7.4, as verified with a pH meter (Mettler Toledo, Madrid, Spain). The medium was prepared with citric acid (0.05 M) and sodium bicarbonate (0.18 M) to adjust the desired pH level. For the tests, a portion of each hydrogel was cut and its initial weight recorded. The sample was then submerged in 20 mL of the prepared aqueous medium at 37 °C. The change in weight was followed by gravimetry. The weight of the hydrogel was recorded every 10 min for the first hour and every 30 min for the next 2 h and finally at 24 and 48 h. The procedure was performed by triplicate. Data collection was interrupted if an asymptotic change in weight was observed or the sample started to degrade [63]. The percentage of swelling was determined according to Equation (2).

(2) $$W_{c}=\frac{W_{S}-W_{D}}{W_{D}}\cdot 100,$$

where *W_C_* is the hydration percentage, *W_S_* is the gel’s weight after swelling and *W_D_* is the weight of the xerogel.

### 2.9. Rheological Response

The rheological analyses were carried out in a Discovery Series Hybrid Rheometer-1 (TA Instruments, New Castle, DE, USA) by running a frequency scan between 0.1 and 100 Hz at a constant amplitude of 1.0% strain and 25 °C [64]. A parallel-plate (diameter 20 mm) geometry was used with a fixed gap distance (1.0 mm) between the plates [14,65]. Samples for the analysis were obtained with the aid of a hollow punch with 1 mm thickness and 20 mm diameter.

### 2.10. Thermal Stability Analyses

Thermogravimetric analyses (TGA) were conducted in the range of 20 to 800 °C to estimate the thermal stability of the hydrogels. Differential scanning calorimetry (DSC) analyses were implemented in the same temperature range to determine the amount of heat absorbed or released by the polymeric material when heated at a constant rate (10 °C/min) [66]. TGA was carried out for a sample of about 20 to 40 mg under a controlled atmosphere with 100 mL/min ultra-high purity Nitrogen (UHP). The impact of pH on the thermal stability of the hydrogels was also analyzed via TGA in the range of 20 to 500 °C. The hydrogels were evaluated by collecting thermograms before and after treatment with solutions at different pH values (see below for details). This was also the case for hydrogels tested in the milliliter scale bioreactor (see below for more information). The instrument used for the tests was the Q600 Simultaneous TGA/DSC (TA Instruments, New Castle, DE, USA).

### 2.11. Mechanical Resistance Evaluation

The bloom strength of the hydrogels was determined according to the International Standard (ISO/DIS 9665 Adhesives, Animal glues, Methods of sampling and testing) with the aid of a TA.HDplusC Texture Analyzer (Stable Micro Systems, Godalming, UK). The tests measured compression at a 0.5 mm/s speed and 4.0 mm distance. Measurements were conducted for hydrogels formed in glass containers by maintaining the same fluid height at room temperature for all treatments. Also, variations in the pH values of the solution were performed for this characterization by preparing buffers at different pH values (3.0, 5.0, 7.0 and 9.0). The adjustment of pH was achieved with solutions of HCl 37% and NaOH solid. The hydrogels were submerged in the solutions for 72 h at 30 °C, which corresponds to the milliliter-scale bioreactor operation conditions. Also, hydrogels samples were maintained in the bioreactor for 72 h at the same temperature, volume, medium and aeration conditions. After bioreactor operation, the hydrogels were taken out to conduct a firmness test to evaluate changes in the mechanical response. This test measures force in compression at a 1.0 mm/s speed and 5.0 mm distance using the same Texture Analyzer described above.

### 2.12. Performance of Encapsulates in a Milliliter Scale Bioreactor 

The encapsulates with the yeast cells were packed in a milliliter scale (250 mL), external-loop airlift-bioreactor to test their performance. The system was designed and assembled in-house by 3D printing (Stratasys, USA) the base in polylactic acid (PLA) while manufacturing the body and lid from commercially available polypropylene. The external loop and connectors were cast in silicone rubber using 3D printed molds. A schematic picture of the setup is shown in Figure S2 (in Supplementary Materials). Before the operation, all the parts were autoclaved and subsequently assembled in a laminar flow hood. Next, aseptically, 15 half-sphere hydrogels (5.0 mL volume each one, Figure S2) were placed in the reactor and the YNB liquid medium, enriched with lactose (16% (w/v)) was added to reach a 250 mL operation volume. This configuration resulted in a 30/70 ratio of solid material to the liquid medium or equivalent to a 70% void fraction. This allowed proper agitation of the packed material and prevented agglomerations that can ultimately lead to dead mass-transfer zones along with the flow pattern of the ascending gas. The system was maintained at 30 °C with aeration provided by an air pump (AC9904 RESUN, 8W) for 72 h. The samples were collected every 3 h and stored at −20 °C until further use.

### 2.13. Chromatography Analysis

The concentration of lactose, glucose, ethanol, lactic acid, acetic acid and glycerol present in the reaction media was estimated with the aid of a High-Performance Liquid Chromatography system (HPLC), 1260 Infinity (Agilent Technologies, Santa Clara, CA, USA), equipped with an Aminex HPX-87H column (Bio-Rad), which is useful for the detection of organic acids, sugars and alcohols. The mobile phase was 5 mM sulfuric acid (solution with type I water) at an elution rate of 0.6 mL/min. Runs with standards were performed to verify the reproducibility of retention times of the compounds of interest. If the peaks observed in the RID/DAD detector were not sufficiently resolved, the samples were diluted until reaching acceptable accuracy.

### 2.14. Performance of Encapsulates in the Simulated Gastrointestinal Tract Media 

A single half-sphere hydrogel was placed in a 250 mL flask with 100 mL of different solutions simulating the conditions of saliva, stomach and small intestine. The simulated saliva medium was prepared according to the work of Li, et al. [67], with slight modifications and contained 1.4 mg/mL NaCl, 0.5 mg/mL KCl, 0.1 mg/mL CaCl_2_, 0.15 mg/mL NaH_2_PO_4_, 0.025 mg/mL MgCl_2_, 0.09 mg/mL CO(NH_2_)_2_, 0.2 mg/mL C_6_H_12_O_6_, 2.5 units/mL *α*-amylase, 0.7 units/mL lysozyme and pH adjusted to 7.0 a with solid NaOH. The stomach and intestine simulated media were based on the work described by Klein and collaborators [68] with slight modifications. The simulated stomach medium was prepared with 80 μM C_24_H_39_NaO_5_, 0.16 mg/mL egg lecithin, 34.2 mM NaCl and pH adjusted to 2.0 with a solution of HCl 37% (PanReac AppliChem, Spain). Finally, the small intestine was simulated with a medium containing 3 mM C_24_H_39_NaO_5_, 1 mg/mL egg lecithin, 68.6 mM NaCl, 19.12 mM C_4_H_4_O_4_ and pH adjusted to 7.0 a with solid NaOH. The treatment began by exposing the hydrogel to the simulated saliva medium for 7 min, then to the simulated gastric fluid medium for 2 h and finally to the small intestine medium for two more h. The whole process was performed with incubation at 37 °C and 150 RPM [8,34].

## 3. Results and Discussion

### 3.1. Morphological Structure and Cells Encapsulated

**Figure 2:**
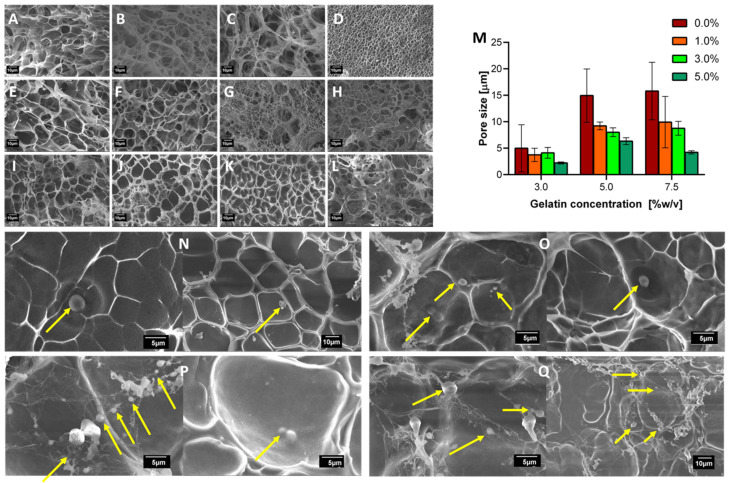
Surface morphology described by scanning electron microscope (SEM) micrographs of crosslinked and uncrosslinked hydrogels in the presence and absence of yeast cells. First row is for 3.0% (w/v) gelatin concentration and varying GTA concentrations from 0.0% (**A**), 1.0% (**B**), 3.0% (**C**) to 5.0% (w/w) (**D**). Second row is for 5.0% (w/v) gelatin concentration and varying GTA concentrations from 0.0% (**E**), 1.0% (**F**), 3.0% (**G**) to 5.0% (w/w) (**H**). Finally, 7.5% (w/v) gelatin concentration and varying GTA concentrations from 0.0% (**I**), 1.0% (**J**), 3.0% (**K**) to 5.0% (w/w) (**L**). (**M**) Average pore size for all treatments. 7.5% (w/v) gelatin, 3.0% (**N**) and 5.0% (**P**) (w/w) GTA hydrogel, before bioreactor operation. Moreover, micrographs after 72 h bioreactor operation are presented for 3.0% (**O**) and 5.0% (**Q**) (w/w). The yellow arrows point to dehydrated *K. lactis* cells compartmentalized into the gel pores.

The analyses and the collected images shown in Figure 2A–L confirmed that, in general, an increase in the concentration of glutaraldehyde leads to a decrease in the average pore size of the gel. Under such circumstances, it is likely that the polymer network becomes more compact and thereby exhibiting higher mechanical strength, as described elsewhere [69]. Figure 2M shows that the average pore size for each treatment as calculated from the scanning electron microscopy (SEM) micrographs. This a critical parameter to determine whether the encapsulated yeast cells can reside and thrive within the matrix. Adequate pore size distributions allow the diffusion of required substrates such as polyacrylamide and polyethylene glycol, and, consequently, high viability levels [70,71].

The found surface morphologies confirmed the effectiveness of the crosslinking strategy via glutaraldehyde. However, it is imperative to note that at the highest concentration of gelatin, the heterogeneity of the gel matrix significantly increased. This is evidenced by the considerable variability in pore size distribution. This is most likely due to insufficient glutaraldehyde to carry out crosslinking reactions. According to the average size of the *K. lactis*, the hydrogels selected to continue with the encapsulation were those that exhibited an average pore size between 3 and 8 µm.

Yeast cells were encapsulated in the selected hydrogels with an average EE of 10%, as calculated using Equation (1) and subsequently imaged via SEM. The micrographs allowed direct visualization of cells fixed on the hydrogel surface. Figure 2N–Q shows that the hydrogel pores are likely to act as microchambers to house the cells that are initially incorporated. These essential spaces are of the utmost importance for survival and even proliferation during subsequent incubation processes. Figure 2N–Q also strongly indicates that a cell network is formed on the surface of the hydrogels after the operation in the milli-bioreactor. This could be explained by the presence of biofilm and aggregation inducing compounds in the medium, such as the GPI-anchored cell surface glycoprotein, which is essential for the pseudohyphal formation and invasive growth [72]. The micrographs also point to a decrease in the average cell size (see yellow arrows in Figure 2N–Q), which could be related to the dehydration of hydrogels in the liquid nitrogen treatment required before imaging.

### 3.2. Functional Groups Identification

**Figure 3:**
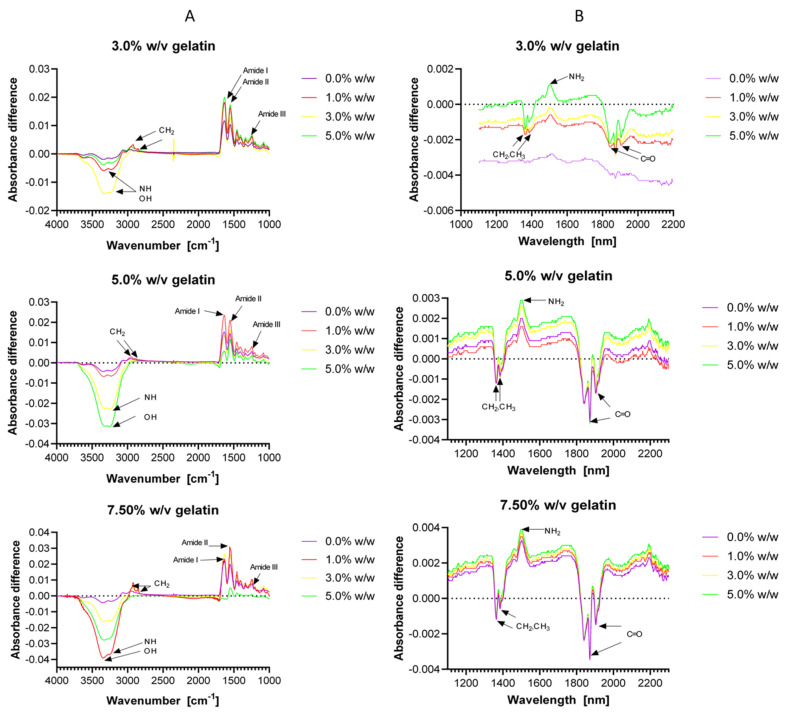
Fourier transform infrared (FTIR) (**A**) and near-infrared (NIR) (**B**) spectra obtained for hydrogels formulations with 3.0%, 5.0% and 7.5% (w/v) gelatin and for concentrations of GTA of 0.0%, 1.0%, 3.0% and 5.0% (w/w). Some relevant peaks are indicated in the spectra for the precise identification (see discussion) of the material functional groups.

Chemical bonding of hydrogels was evaluated spectroscopically by Fourier transform infrared (FTIR) and near-infrared (NIR) spectroscopies. Typical FTIR absorption bands amide I, amide II and amide III of gelatin [73] were found at 1630 and 1631 cm^−1^ (carbonyl group C=O [74]), 1550 and 1552 cm^−1^ (C-N bond) and 1245 cm^−1^ (N-H vibrations [75]), respectively (Figure 3A). The small peaks observed between 2940 and 2850 cm^−1^ can be associated with asymmetric and symmetric stretching vibration of the CH_2_ bond [73] (Figure 3A). This bond is present along the gelatin backbone and is also formed by the crosslinking reaction. The peaks protruding between 3340 and 3220 cm^−1^ can be associated with the N-H bond of the free terminal amine groups of the gelatin (Figure 3A). This indicates that some of the gelatin groups involved in crosslinking remained unreacted. This band can also be assigned to the O-H groups of water present in the hydrogel [74].

Figure 3B shows NIR peaks between 1364 and 1384 nm, which are associated with the CH_2_ and CH_3_ groups present along the backbone of the polymer network. The single peak at about 1500 nm is directly related to the free amine groups (NH_2_) of gelatin and confirms incomplete crosslinking. The successive peaks observed between 1840 and 1904 nm are for C=O bonds, which are likely due to the amide bonds of gelatin or excess glutaraldehyde [76,77,78]. The small differences observed for both FTIR and NIR spectra suggest no significant differences in the chemical structure of the material for the evaluated glutaraldehyde concentrations. Importantly, the peaks intensity is slightly altered by the gelatin concentration in all the range spectrum studied.

### 3.3. Hydrogels Swelling Degree 

**Figure 4:**
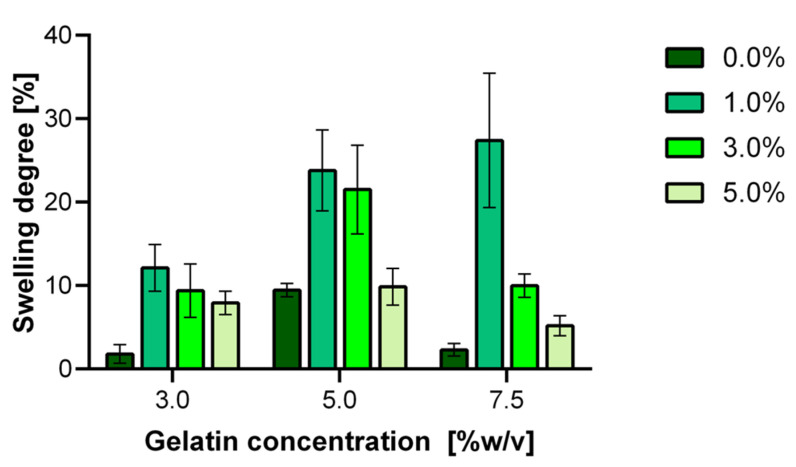
Swelling behavior of hydrogels prepared with varying levels of gelatin and degrees of crosslinking. The experiments were conducted in aqueous solution at pH 7.4 and 37 °C.

Hydrogels were subjected to swelling in an aqueous medium at pH 7.4 and 37 °C. Figure 4 shows the degree of swelling for the different gelatin and GTA concentrations. For all gelatin concentrations, the swelling degree increases for 0.0 to 1.0% (w/w) GTA to reach a maximum and then steadily decreases for the 3.0 and 5.0% (w/w) GTA treatments. We hypothesize that this maximum is most likely attributed to a matrix that provides a sufficient level of freedom and permeability to allow a significant penetration of water molecules to an environment where they remain trapped. As the crosslinking degree is increased, the polymer network becomes more compact and, therefore, with a limited capacity to withstand water molecules. This behavior agrees with previous reports for similar hydrogels [63]. Further testing will be required to confirm these notions. The maximum degree of swelling appears to increase from about 10% at 3.0% (w/v) gelatin to about 25% and 30% for the 5.0% and 7.5% (w/v) gelatin, respectively. This is most likely a consequence of a larger number of gelatin chains available for interaction with water molecules and the larger pore size observed for these materials (Figure 4).

As expected, complete degradation for uncrosslinked hydrogels made at 3.0% (w/v) gelatin takes about 30 min. This can be attributed to the loose and porous structure of the hydrogel, where water molecules can freely penetrate to dissolve the gelatin chains eventually. Once the matrix is destabilized, we obtained a viscous liquid suspended in the aqueous media. We observed a somewhat superior structural stability for uncrosslinked hydrogels made at 7.5% (w/v) compared with the lower gelatin concentrations. This is evidenced by a longer total degradation time of about six days. These results agree well with previous reports for hydrogels in aqueous media [71,79,80].

### 3.4. Hydrogels Rheological Behavior

**Figure 5:**
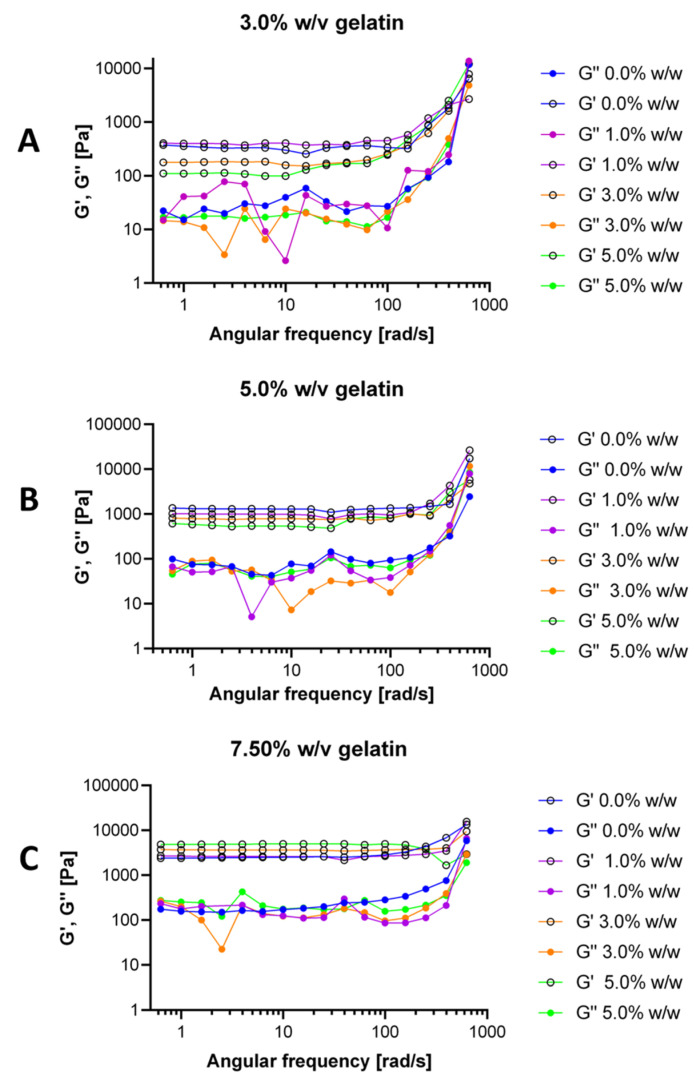
Storage and loss moduli for 3.0% (**A**), 5.0% (**B**) and 7.5% (w/v) (**C**) gelatin and for concentrations of GTA of 0.0%, 1.0%, 3.0% and 5.0% (w/w).

As shown in Figure 5, the oscillatory tests reveal that, in general, the storage moduli (G’) are higher than the loss moduli (G”) for all treatments. This shows that crosslinking induced a typical solid-like gel behavior [81,82]. We observed occasional inversion of this trend for very high frequencies. This indicates that the material changes from solid to a fluid as it is subjected to high oscillatory stress. Accordingly, it appears that physical interactions between the chains in the network are dominant [82]. The dominance of the storage moduli confirms that the elastic response is the one that dominates in the hydrogels, which also provides further evidence of a stable structure and the ability of the gels to store deformation energy in an elastic manner [64]. G’ and G” increased when the frequency was increased, thereby indicating that the material became stiffer at higher frequencies [83]. Conventionally, a fully cured tridimensional network presents G’ curves with a constant slope and independent of the angular frequency [84,85]. As shown in Figure 5, this was only the case for our 7.5% (w/v) gelatin hydrogels, which could be attributed to a more homogeneous three-dimensional matrix compared with the other formulations. This rheological behavior strongly suggests that the added crosslinking agent failed to react with the gelatin chains completely. As a result, the obtained matrix exhibits some isolated regions where gelatin keeps its original configuration.

**Figure 6:**
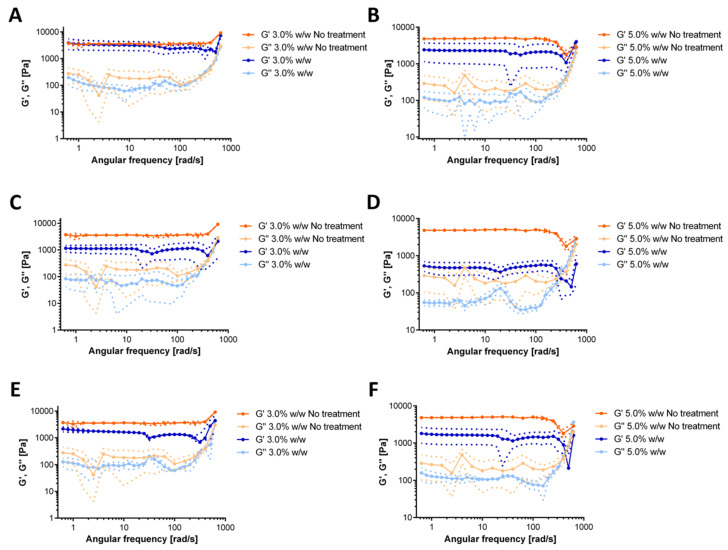
Storage and loss moduli for hydrogels after exposure to gastrointestinal tract simulated media and the comparison with a hydrogel in the absence of the treatment. Saliva simulated medium Figure 3. 0% (w/w) (**A**) and 5.0% (w/w) (**B**) GTA concentration. Stomach simulated medium for 3.0% (w/w) (**C**) and 5.0% (w/w) (**D**) GTA concentration. Small Intestine simulated medium for 3.0% (w/w) (**E**) and 5.0% (w/w) (**F**) GTA concentration.

The rheological response of the hydrogels after exposure to each simulated gastrointestinal tract media is shown in Figure 6. For the simulated saliva treatment, the 3.0% (w/w) GTA showed no significant changes in the moduli concerning the control (Figure 6A). In the case of the 5.0% (w/w) GTA, the variation is less subtle, and we identified a slight reduction in both moduli after treatment (Figure 6B). This likely indicates a subtle reduction in the structural stability of the gel. Exposure to the simulated stomach medium led to a significant decrease in both moduli. These reductions reached about five-fold in the case of the 3.0% (w/w) GTA (Figure 6C) and of about ten-fold for the 5.0% (w/w) GTA (Figure 6D). Once again, this reflects marked altered structural stability and particularly the detrimental impact of low pH conditions. Finally, upon exposure to the small intestine medium, changes in the rheological behavior were insignificant for the 3.0% (w/w) GTA (Figure 6E), while a two-fold reduction was observed for the 5.0% (w/w) GTA (Figure 6F). Severe alterations of the structure were only observed at very high frequencies of oscillation, which are not expected during the regular pass through the human gastrointestinal (GI) tract. Importantly, taken together, these results indicate that during the pass through the GI tract, the material will continue to exhibit a solid-like rheological response, which is critical to assure that a large population of probiotics effectively reach the site of action. These results agree well with previous observations of similar encapsulates [86].

**Figure 7:**
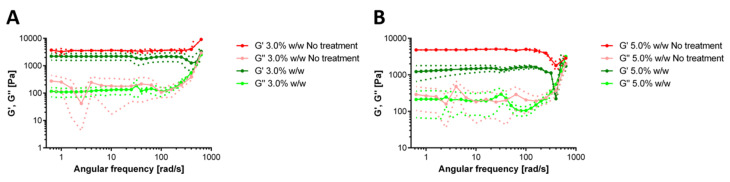
Storage and loss moduli for hydrogels after 72 h of milli-bioreactor operation and the comparison with a hydrogel without this treatment. 3.0% (w/w) (**A**) and 5.0% (w/w) (**B**) gelatin and for concentrations of GTA of 0.0%, 1.0%, 3.0% and 5.0% (w/w).

We also evaluated the rheological response of the hydrogels after the operation in the milli-bioreactor for 72 h (Figure 7). The dominance of the storage modulus over the loss modulus confirms that the elastic response is sufficient to maintain a solid-like structure. After the operation, the 3.0% (w/w) GTA showed no significant changes in the moduli concerning the control (Figure 7A). In the case of the 5.0% (w/w) GTA, there is a notorious effect on the rheological properties, as evidenced by a decrease of about five-fold for the storage module and up to two-fold for the loss modulus (Figure 7). Additionally, the loss modulus crossed the storage modulus at very high frequencies, which indicates possible structural rearrangements during the 72 h of the continuous operation in the bioreactor. These results are comparable with those recently reported for a hydrogel-packed bioreactor [87,88].

### 3.5. Mechanical Resistance Evaluation 

**Figure 8:**
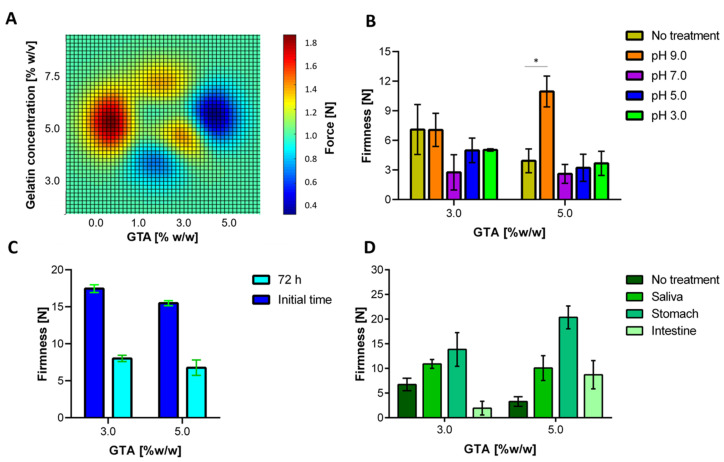
Evaluation of the mechanical response of hydrogels. (**A**) Breaking force for chemically crosslinked hydrogels. The subsequent results are related just to the two treatments selected for cell encapsulation. (**B**) Hydrogel firmness after exposure to media with different pH values. (**C**) Hydrogel firmness after the operation in the milli-bioreactor for 72 h. (**D**) Hydrogel firmness for the gastrointestinal tract simulated application.

Figure 8A shows a contour plot with the obtained breaking force for hydrogels prepared at different gelatin and GTA concentrations. Bloom test results indicated that an increase in the crosslinking agent concentration led to more stable and elastic hydrogels. However, such stiffness increase promoted an increment in the tendency to fracture in the presence of plastic deformation. This behavior has been observed in ceramic materials such as bricks and glasses [89]. This behavior is somewhat counterintuitive as we expected that highly crosslinked hydrogels exhibited the highest resistance to rupture [13,14,15,16,17,18,19,20,21,22,23,24,25,26,27,28,29,30,31,32,33,34,35,36,37,38,39,40,41,42,43,44,45,46,47,48,49,50,51,52,53,54,55,56,57,58,59,60,61,62,63,64,65,66,67,68,69,70,71,72,73,74,75,76,77,78,79,80,81,82,83,84,85,86,87,88,89,90]. This could be attributed to the unevenness of the crosslinking reaction throughout the polymer matrix, which is, in turn, related to increasingly higher mass transfer limitations as the gel is formed. This leads to localized and isolated changes within the 3D structure of the matrix. As a result, induced fractures propagate quickly on crosslinked materials when compared with the uncrosslinked counterparts, where due to higher cohesiveness, resistance is superior. Similar results were observed by Markov et al. while exploring the mechanical properties of pectin and calcium chloride hydrogels [91].

Figure 8B shows that, concerning the control, the hydrogel firmness is independent of the pH of the medium, with the only exception of pH 9.0. In this case, the firmness is equal (3.0% (w/w) GTA) or improves (5.0% (w/w) GTA) with respect to the control. This could be explained by the crosslinking of the polymeric chains mediated by free cations in the medium, such as sodium (Na). These crosslinking-side reactions appear to compensate for the firmness loss due to swelling. However, in absolute terms, there is less degradation in the most acidic environments. Apart from that, to inquire about a more accurate approximation, hydrogels were packed into the milli-bioreactor. Under these conditions, there was a significant difference in firmness concerning the control level, as is presented in Figure 8C. As a result, we can conclude that the fermentation conditions (e.g., medium characteristic and aeration) promoted material degradation, thereby leading to half of the original firmness for both cases. Finally, Figure 8D shows that hydrogels’ firmness increases as the two materials are exposed to the first two simulated gastrointestinal tract media, that is, saliva and stomach. This is most likely due to the incorporation of various salts from the saliva medium into the matrix. The further increase in firmness observed for the low pH stomach medium can be attributed to the protonation of the pendant amine groups of gelatin backbone chains, which promote increased ion interaction that toughens the surface of the matrix. As a result, even though the elastic response is maintained, penetration requires a higher strength. We also observed a reduction in firmness for the small intestine simulated media. This could be explained by the neutral pH of the medium that is likely to promote the degradation of the material. This is favorable since the polymeric material will be likely to overcome pass through the stomach without changing its structural stability but becomes unstable and penetrable at the intestine where the release of the probiotic cells is desirable.

### 3.6. Thermal Resistance Evaluation

Thermogravimetric analyses were conducted in the range of room temperature to 800 °C to estimate the thermal stability of the hydrogels. The collected thermograms showed an initial weight loss of about 100 °C, which can be correlated to the water in the sample. This was followed by the decomposition of the gelatin-glutaraldehyde network, which presented high resistance up to 300 °C, in all cases. The weight derivative confirms these observations as it shows a sharp peak with a maximum at around 100 °C and small changes above 300 °C as shown in Figure S3 (in Supplementary Materials). The weight loss appears to be accelerated for the hydrogels without crosslinking.

**Figure 9:**
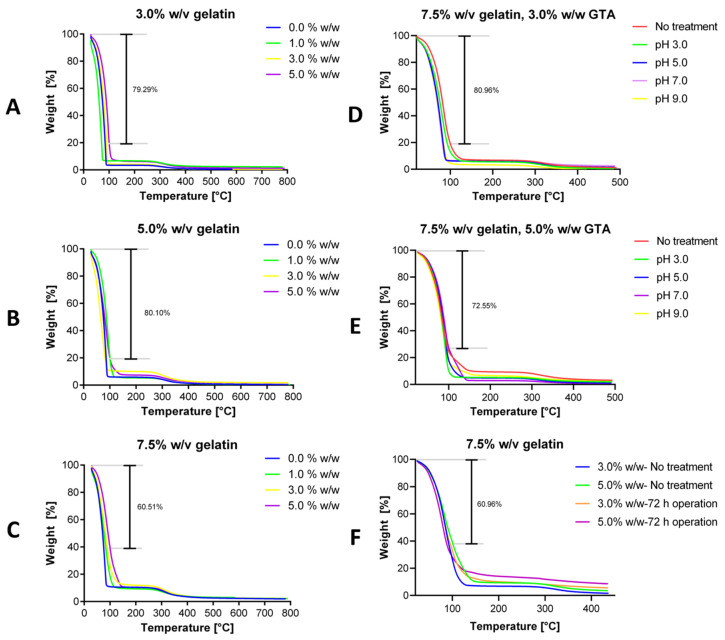
The minimum weight (%) loss at 100 °C is indicated on each plot. Thermograms for hydrogels with 3.0% (**A**), 5.0% (**B**) and 7.5% (**C**) (w/v) gelatin concentration. GTA concentrations of 0.0% (w/w) (blue), 1.0% (w/w) (green), 3.0% (w/w) (yellow) and 5.0% (w/w) (purple). Thermal degradation after exposure to different pH media for 3.0% (**D**) and 5.0% (w/w) (**E**) GTA hydrogels and after the milli-bioreactor operation (**F**).

Moreover, the results suggest an increase in thermal resistance for gels with higher crosslinking levels, as evidenced by the smaller weight loss at 100 °C compared with hydrogels with lower crosslinking degrees. The uncrosslinked samples show the most deficient stability in all the cases and those with higher crosslinking degrees present the minimum weight loss, which is explicit in Figure 9A–C. Additionally, by increasing the gelatin concentration, the thermal stability at about 100 °C improves considerably. This could be attributed to a higher amount of free material that could hold water more efficiently or that a more significant gelatin concentration could increase the chain entanglement [92]. As observed in Figure 9A–C, after increasing the gelatin concentration from 3.0% to 7.5% (w/v), the hydrogel can retain up to twice as much of the encapsulated matter. From these results, it is evident that the crosslinked gelatin hydrogels are highly resistant at least up to 270 °C [93]. This fact is undoubtedly exciting; however, most bioprocesses operate between 30–50 °C, which ensures that for most applications, the thermal degradation of the developed hydrogels will be minimal.

Figure 9D,E show the thermal stability of 3.0% and 5.0% (w/w) GTA hydrogels after exposure to media at different pH values. For the case of 3.0% (w/w) GTA, an increase in pH led to a decrease in thermal resistance between 5.98% and 14.53% at 100 °C and between 0.34% and 3.63% at 270 °C, compared to hydrogel without the pH treatment. Similar results were found for the 5.0% (w/w) GTA case with a decrease between 6.48% and 14.22% at 100 °C and between 2.96% and 6.23% at 270 °C. Treatments for pH 7.0 and above led to the most significant losses in thermal stability. A possible explanation for this behavior can be found in the ionization of the carboxyl groups in alkaline pH by the ionic repulsion of the protonated carboxyl groups and by the potential interaction between the hydroxyl ions present in the medium and the pendant amine groups of gelatin backbone. As the amine groups are deprotonated, cations in the medium move to the backbone to balance charges. The presence of these cations might decrease the stability of the network by structure bonding alteration [80]. Figure 9F shows the thermal stability of 7.5% (w/v) gelatin hydrogels after 72 h of operation in the milli-bioreactor. The resistance to thermal degradation increases for both the 3.0% and 5.0% (w/w) GTA hydrogels after 72 h under the conditions of the bioreactor operation. For the case of 3.0% (w/w) GTA, the bioreactor operation led to an increase in thermal resistance of 6.64% at 100 °C and 2.46% at 270 °C, compared to the control. Similar results were found for the 5.0% (w/w) GTA case, where at 100 °C, the thermal resistance increased by 10.90% and by 3.91% at 270 °C. This could be explained due to hydrogel swelling capacity and by the incorporation of water, salts and large molecules such as sugars from the fermentation medium into the matrix [94]. Also, some of the extracellularly secreted metabolites might have accumulated into the polymer matrix, increasing the total weight of the hydrogel.

### 3.7. Proof-of-Concept: Milli-Bioreactor Operation

**Figure 10:**
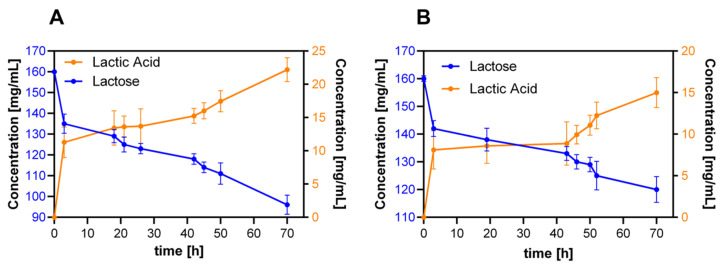
Proof-of-concept: milli-bioreactor production by packing 7.5% (w/v) gelatin hydrogels made with 3.0% (**A**) and 5.0% (**B**) (w/w) GTA concentration.

Initially, batch bioprocesses were conducted with the hydrogels selected for probiotic cell encapsulation at 1.5% (w/v) biomass concentration and with culture medium supplemented with lactose 2.0% or 4.0% (w/v). Under these conditions, the production of metabolites was directed towards acetic acid, glycerol and almost none lactic acid. The second run showed that, by increasing four times the lactose (substrate) available in the medium, the lactic acid production was favored even under aerobic operation. We hypothesize that this is likely because the high gradient concentration induces diffusion into the porous material, thereby facilitating a metabolic pathway towards lactic acid. Besides, the almost invariable glucose concentration observed during the experiment suggested that the primary carbon source for this strain is indeed lactose. With this background in mind, we decided to increase the biomass to concentration to 5.0% (w/v) and to maintain the lactose concentration constant at 16% (w/v). The results of metabolite production are shown in Figure 10. The first observation is that the sampling time should be extended two- to three-fold due to the relatively high remaining substrate concentration, that is, 121 and 95 mg/mL for the 3.0% and 5.0% (w/w) GTA hydrogels, respectively. The final conversion efficiencies for the tested formulations approach 37.5% and 35.4%. This clearly illustrates the moderate ability of the packed system to transform the fermentable sugar (lactose) into lactic acid under aerobic conditions.

**Figure 11:**
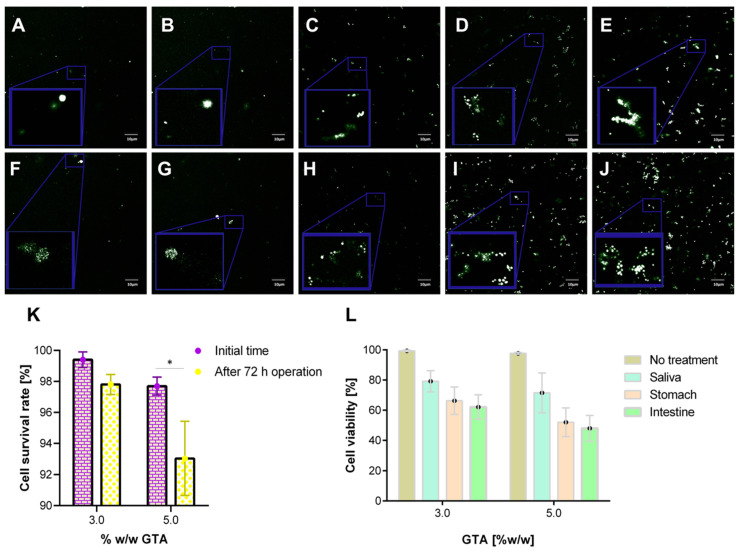
Confocal microscopy images. Dead cells are shown in white color while live cells in green. Scale bar corresponds to 10 μm. (**A**) Live/dead *K. lactis* cells in the encapsulates made with 3.0% (w/w) GTA. (**B**) Live/dead *K. lactis* cells in the encapsulates made with 3.0% (w/w) GTA after 72 h of bioreactor operation. (**C**) Live/dead *K. lactis* cells in the encapsulates made with 3.0% (w/w) GTA after exposure to simulated saliva medium. (**D**) Live/dead *K. lactis* cells in the encapsulates made with 3.0% (w/w) GTA after exposure to simulated stomach medium. (**E**) Live/dead *K. lactis* cells in the encapsulates made with 3.0% (w/w) GTA after exposure to the simulated small intestine medium. (**F**) Live/dead *K. lactis* cells in the encapsulates made with 5.0% (w/w) GTA. (**G**) Live/dead *K. lactis* cells in the encapsulates made with 5.0% (w/w) GTA after 72 h of bioreactor operation. (**H**) Live/dead *K. lactis* cells in the encapsulates made with 5.0% (w/w) GTA after exposure to simulated saliva medium. (**I**) Live/dead *K. lactis* cells in the encapsulates made with 5.0% (w/w) GTA after exposure to simulated stomach medium. (**J**) Live/dead *K. lactis* cells in the encapsulates made with 5.0% (w/w) GTA after exposure to the simulated small intestine medium. In all images, the inset corresponds to a zoom of a region of interest. Yeast probiotic cell survival rate for encapsulates before and after bioreactor operation (**K**) and after treatment with each of the gastrointestinal tract simulated media (**L**).

The unusually low efficiency of the 5.0% (w/w) GTA gels can be explained by the small cell viability measured in this case (see below Figure 11K). Nonetheless, the rate of reaction is higher in the 3.0% (w/w) GTA case, as the same final concentration obtained with the 5.0% (w/w) GTA hydrogel is reached in about half the required time. This is most likely due to the larger pore size of 3.0% (w/w), which largely avoids any possible mass transfer limitations. As a result, the substrates might diffuse freely into the porous matrix, thereby favoring cell survival and, consequently, superior metabolic activity.

### 3.8. Cell Viability Assays

Encapsulation of *K. lactis* proceeded with hydrogels containing 7.5% (w/v) gelatin made with either 3.0% or 5.0% (w/w) GTA. The results indicated that the encapsulating material is highly biocompatible for cell culture and also demonstrated the high resilience of this strain during bioreactor operation. The confocal images in Figure 11A,B,F,G show a significantly lower number of dead cells in the packed hydrogels both before and after the milli-bioreactor operation for 72 h. A quantitative analysis of the images demonstrated that for 3.0% (w/w) GTA, the viable cells were reduced in about 2%, while for the 5.0% (w/w) GTA, the reduction approached 5% (Figure 11K). This difference is most likely due to the excess of unreacted GTA for the 5.0% (w/w) GTA hydrogels, which has been reported to be highly cytotoxic [59,95]. Additionally, due to the reduced pore size, in this case, mass transfer limitations and restricted space for proliferation are likely to play a significant role.

The 3.0% or 5.0% (w/w) GTA hydrogels with encapsulated yeast cells were also exposed to simulated saliva, stomach and small intestine media. The confocal images in Figure 11C–E show a progressive reduction in cell viability as the 3.0% (w/w) GTA encapsulates are exposed to the simulated media. We found similar results for the 5.0% (w/w) GTA encapsulates (Figure 11H–J). A quantitative analysis of the images demonstrated that for 3.0% (w/w) GTA, the viable cells were reduced in about 20%, 35% and 40% for simulated saliva, stomach and small intestine media, respectively (Figure 11L). A similar analysis for the 5.0% (w/w) GTA showed a reduction of about 30%, 50% and 55% (Figure 11L). As for the hydrogels after bioreactor operation, the differences observed here might be related to mass transfer issues and restricted proliferation. These results are consistent with those obtained for the encapsulates of mouse embryonic fibroblast 3T3 cells in gelatin hydrogels [96] and fibroblasts in gelatin/chitosan hydrogels [97]. The fact that about 50%–60% of the encapsulated cells remain active when reaching the intestine is encouraging to continue working on the development of novel probiotic encapsulates from gelatin matrices.

## 4. Conclusions

A polymeric chemically-crosslinked hydrogel of natural origin, namely, collagen, was successfully synthesized, characterized and tested for its applicability in the encapsulation of yeast cells. We characterize the hydrogels with the aid of microscopy, spectroscopy, thermal and mechanical techniques. SEM images revealed that pore size is a strong function of the crosslinking agent, glutaraldehyde (GTA) and the contents of collagen. Additionally, swelling and rheological behavior suggested that high collagen and GTA contents led to materials capable of maintaining structural stability even under extreme operating conditions. This was confirmed by firmness and thermal stability analysis, which provided an even ampler regime of operating conditions that included firmness of up to 10 N and temperatures of up to 270 °C. Spectroscopy methods allowed us to verify that even though chemical crosslinking was partially completed, it was sufficient to preserve the required solid-like behavior for the hydrogels. Based on these results, we selected 7.5% (w/v) gelatin contents and 3% and 5% (w/w) GTA as suitable for cell encapsulation.

Packings of semi-spherical topologies with encapsulated *K. lactis* probiotic cells were prepared and tested within milli-bioreactors and gastrointestinal tract simulated media. In the first case, the encapsulates were packed in the bioreactor and we measured the production of lactic acid and cell viability after 72 h operation. In the best case, we managed to produce only 100 mg/mL of lactic acid with efficiencies below 40%. This was attributed to the aerobic operating conditions and, to some extent, to possible mass transfer limitations. Cell viability after operation remained above 95%, which is encouraging for further optimization. In the second case, subsequent pass through simulated gastrointestinal (GI) media only led to a reduction of viability in the hydrogels by about 50%. Mechanical, thermal and rheological characterization after bioreactor operation and simulated GI tract media treatments confirmed sufficient integrity and structural stability. These results are appealing when compared with commercially available and recent research reports of encapsulated probiotics. We are confident that the proposed encapsulation strategy is a viable avenue to address some of the main issues of probiotics preparations regarding the stability and ultimate effectiveness of the administered bioactive components.

## Supplementary Materials

The following are available online at https://www.mdpi.com/2073-4360/12/6/1287/s1, Figure S1: Schematic of the mechanism of gelatin crosslinking. A. Gelatin polymeric chain and glutaraldehyde (GTA) structure. The presence of the two highly reactive aldehyde groups in GTA is an essential factor in selecting it as a crosslinker. B. The reaction starts with the nucleophilic addition of the primary amine to the carbonyl group at each end of the GTA molecule. C. Amine deprotonation and oxygen protonation occur. D. By incorporating an H+-ion, the deprotonation of the hydroxyl group is developed. E. Finally, the imine group is formed by protonation of the amine again and the release of water. F. Conceptual model of the encapsulation of probiotic yeasts in the chemically crosslinked gelatin matrix. Figure S2: Packed-bed bioreactor designed and 3D-printed for this study. A. 3D model. B. Isometric view. C. 3D-printed bioreactor with a half-sphere gel sample. D. Silicone molds used to form the gels. E. Final assembly. Figure S3: Weight rate of change with respect to the temperature for hydrogels with 3.0% (A), 5.0% (B) and 7.5% (C) (w/v) gelatin concentration. Thermal degradation after exposure to different pH media for 3.0% (D) and 5.0% (w/w) (E) GTA hydrogels and after the milli-bioreactor operation (F).
