# Supplementary material for: Formulation and Characterization of Gelatin-Based Hydrogels for the Encapsulation of Kluyveromyces lactis—Applications in Packed-Bed Reactors and Probiotics Delivery in Humans
Source: Polymers (Basel). 2020 Jun 4;12(6):1287. doi: 10.3390/polym12061287 (PMC7362005; doi:10.3390/polym12061287)
Supplement: Supplementary file 1 [file polymers-12-01287-s001.pdf]

Supplementary Materials

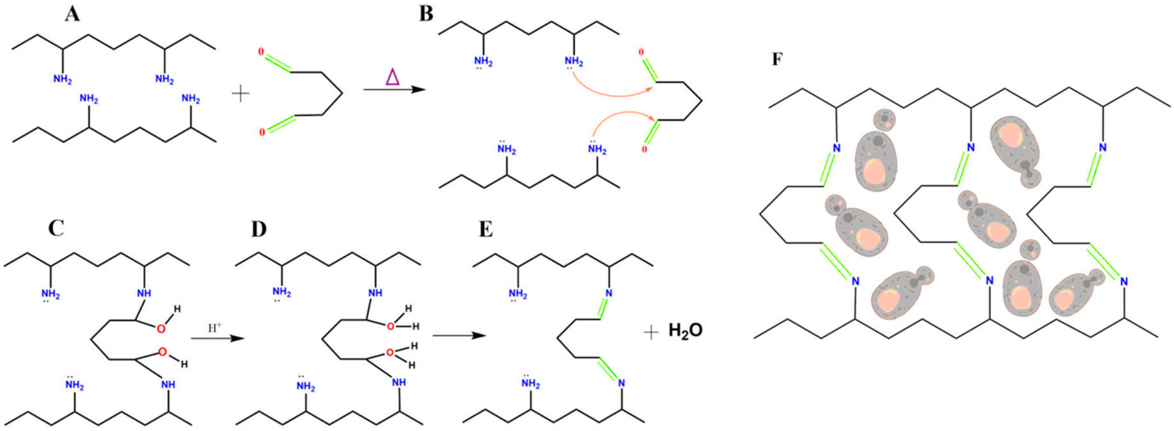

Figure S1. Schematic of the mechanism of gelatin crosslinking. A. Gelatin polymeric chain and glutaraldehyde (GTA) structure. The presence of the two highly reactive aldehyde groups in GTA is an essential factor in selecting it as a crosslinker. B. The reaction starts with the nucleophilic addition of the primary amine to the carbonyl group at each end of the GTA molecule. C. Amine deprotonation and oxygen protonation occur. D. By incorporating an H^+-ion, the deprotonation of the hydroxyl group is developed. E. Finally, the imine group is formed by protonation of the amine again and the release of water. F. Conceptual model of the encapsulation of probiotic yeasts in the chemically crosslinked gelatin matrix.

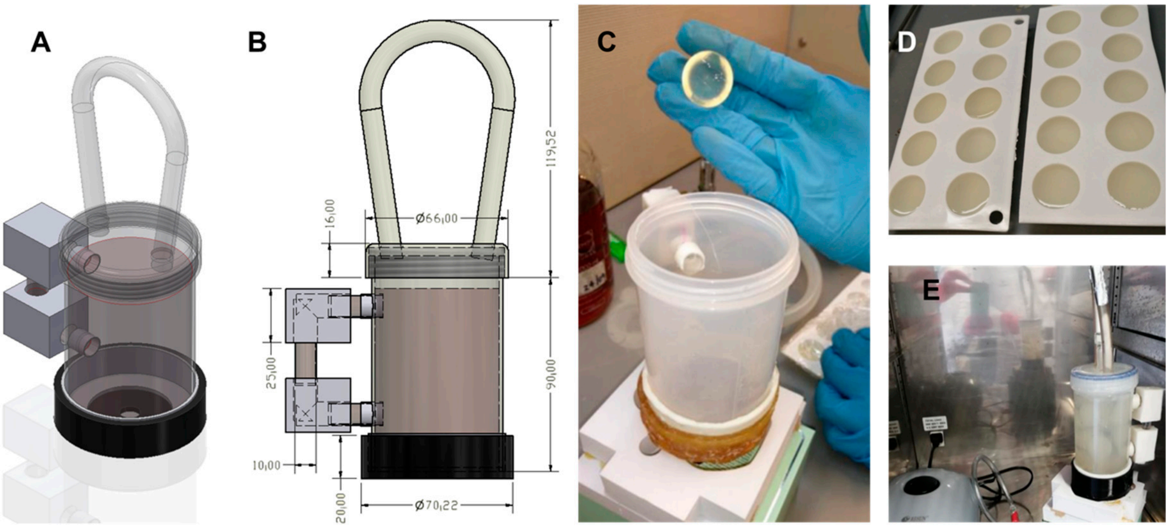

Figure S2. Packed-bed bioreactor designed and 3D-printed for this study. A. 3D model. B. Isometric view. C. 3D-printed bioreactor with a half-sphere gel sample. D. Silicone molds used to form the gels. E. Final assembly.

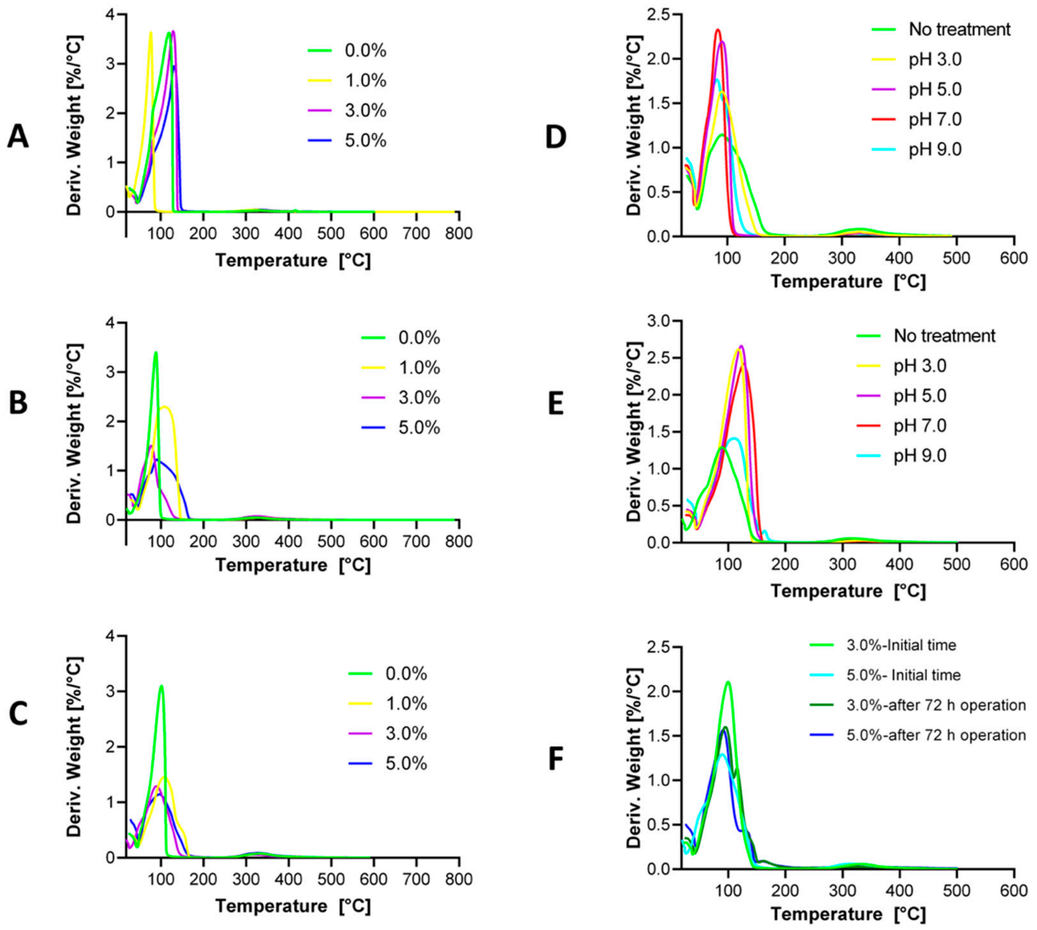

Figure S3. Weight rate of change with respect to the temperature for hydrogels with 3.0% (A), 5.0% (B) and 7.5% (C) (w/v) gelatin concentration. Thermal degradation after exposure to different pH media for 3.0% (D) and 5.0% (w/w) (E) GTA hydrogels and after the milli-bioreactor operation (F).
